# mRNA Degradation Rates Are Coupled to Metabolic Status in Mycobacterium smegmatis

**DOI:** 10.1128/mBio.00957-19

**Published:** 2019-07-02

**Authors:** Diego A. Vargas-Blanco, Ying Zhou, L. Gregory Zamalloa, Tim Antonelli, Scarlet S. Shell

**Affiliations:** a Department of Biology and Biotechnology, Worcester Polytechnic Institute, Worcester, Massachusetts, USA; b Department of Mathematics, Worcester State University, Worcester, Massachusetts, USA; c Program in Bioinformatics and Computational Biology, Worcester Polytechnic Institute, Worcester, Massachusetts, USA; Hebrew University of Jerusalem

**Keywords:** ATP, *Mycobacterium smegmatis*, carbon starvation, hypoxia, mRNA degradation, mRNA stability, stress response, tuberculosis, *Mycobacterium tuberculosis*

## Abstract

The success of Mycobacterium tuberculosis as a human pathogen is due in part to its ability to survive stress conditions, such as hypoxia or nutrient deprivation, by entering nongrowing states. In these low-metabolism states, M. tuberculosis can tolerate antibiotics and develop genetically encoded antibiotic resistance, making its metabolic adaptation to stress crucial for survival. Numerous bacteria, including M. tuberculosis, have been shown to reduce their rates of mRNA degradation under growth limitation and stress. While the existence of this response appears to be conserved across species, the underlying bacterial mRNA stabilization mechanisms remain unknown. To better understand the biology of nongrowing mycobacteria, we sought to identify the mechanistic basis of mRNA stabilization in the nonpathogenic model Mycobacterium smegmatis. We found that mRNA half-life was responsive to energy stress, with carbon starvation and hypoxia causing global mRNA stabilization. This global stabilization was rapidly reversed when hypoxia-adapted cultures were reexposed to oxygen, even in the absence of new transcription. The stringent response and RNase levels did not explain mRNA stabilization, nor did transcript abundance. This led us to hypothesize that metabolic changes during growth cessation impact the activities of degradation proteins, increasing mRNA stability. Indeed, bedaquiline and isoniazid, two drugs with opposing effects on cellular energy status, had opposite effects on mRNA half-lives in growth-arrested cells. Taken together, our results indicate that mRNA stability in mycobacteria is not directly regulated by growth status but rather is dependent on the status of energy metabolism.

## Abstract (precis)

The logistics of tuberculosis therapy are difficult, requiring multiple drugs for many months. Mycobacterium tuberculosis survives in part by entering nongrowing states in which it is metabolically less active and thus less susceptible to antibiotics. Basic knowledge on how M. tuberculosis survives during these low-metabolism states is incomplete, and we hypothesize that optimized energy resource management is important. Here, we report that slowed mRNA turnover is a common feature of mycobacteria under energy stress but is not dependent on the mechanisms that have generally been postulated in the literature. Finally, we found that mRNA stability and growth status can be decoupled by a drug that causes growth arrest but increases metabolic activity, indicating that mRNA stability responds to metabolic status rather than to growth rate *per se*. Our findings suggest a need to reorient studies of global mRNA stabilization to identify novel mechanisms that are presumably responsible.

## INTRODUCTION

Most bacteria periodically face environments that are unfavorable for growth. To overcome such challenges, bacteria must tune their gene expression and energy usage. Regulation of mRNA turnover can contribute to both of these. However, the mechanisms by which mRNA turnover is carried out and regulated remain poorly understood, particularly in mycobacteria.

During infection, the human pathogen Mycobacterium tuberculosis faces not only the immune response and antibiotics but also nonoptimal microenvironments, such as hypoxia and starvation (1, 2). Regulation of mRNA turnover appears to contribute to adaptation to such conditions. A global study of mRNA decay in M. tuberculosis showed a dramatic increase in transcriptome stability (increased mRNA half-lives) in response to hypoxia, compared to that with aerobic growth (3). This suggests that mRNA stabilization contributes to energy conservation in the energy-limited environments that M. tuberculosis encounters during infection. Similar responses have been shown for other bacteria under conditions that slow or halt growth, including carbon deprivation, stationary phase, and temperature shock (4–13). However, the mechanisms responsible for global regulation of mRNA stability in prokaryotes remain unknown.

A conventional model for RNA decay in Escherichia coli starts with endonucleolytic cleavage by RNase E, particularly in 5′-end-monophosphorylated mRNAs (14–16). The resulting fragments are further cleaved by RNase E, producing fragments that are fully degraded by exonucleases, such as polynucleotide phosphorylase (PNPase), RNase II, and RNase R (17, 18). mRNA degradation is coordinated by the formation of a complex known as the degradosome. In E. coli, RNase E serves as the scaffold for degradosomes containing RNA helicases, the glycolytic enzyme enolase, and PNPase (19–23). Other organisms that encode RNase E form similar degradosomes (24, 25). In bacteria lacking RNase E, other endonucleases assume the scaffold function (26–28). Mycobacteria encode RNase E, but efforts to define the mycobacterial degradosome have produced inconsistent results (29, 30). It is unclear if degradosome reorganization or dissolution contribute to the global regulation of mRNA degradation in any bacteria. Interestingly, the importance of degradosome formation in E. coli varies depending on the carbon sources provided, suggesting links between RNA degradation and metabolic capabilities (31). Furthermore, the chaperones DnaK and CsdA associate with degradosomes in E. coli under certain stresses (20, 32, 33).

Global transcript stabilization in stressed bacteria may plausibly result from reduced RNase abundance, reduced RNase activity, and/or reduced accessibility of transcripts to degradation proteins. In E. coli, multiple stressors upregulate RNase R, possibly to mitigate ribosome misassembly (34, 35), and RNase III levels decrease under cold shock and stationary phase (36). Surprisingly, protein levels for most putative RNA degradation proteins in M. tuberculosis remain unaltered under hypoxic conditions (37), suggesting that mRNA degradation is not necessarily regulated at the level of RNase abundance in mycobacteria. However, there is evidence that RNase activity may be regulated. For example, proteins such as RraA and RraB can alter the function of the RNase E-based degradosome in E. coli (38). Translating ribosomes can mask mRNA cleavage sites and stabilize mRNAs (39). In Caulobacter crescentus, subcellular localization of mRNA degradation proteins may affect global mRNA stability (40, 41). Furthermore, in some actinomycetes, PNPase might be regulated by the stringent response alarmones guanosine-3′-diphosphate-5′-triphosphate (pppGpp) and/or guanosine-3′,5′-bisphosphate (ppGpp), collectively referred to as (p)ppGpp (42, 43). Many bacteria synthesize (p)ppGpp in response to energy stress (44–47), in which it generally facilitates adaptation by upregulating stress-associated genes and downregulating those associated with growth (46, 48–52). (p)ppGpp was reported to inhibit the activity of PNPase in two actinomycetes, Streptomyces coelicolor and *Nonomuraea* (42, 43), suggesting that the stringent response may directly stabilize mRNA as part of a broader response to energy starvation.

Another explanation for stress-induced transcript stabilization may be that reduced transcript abundance directly leads to increased transcript stability. mRNA abundance and half-life were reported to be inversely correlated in multiple bacteria, including M. tuberculosis (3, 8, 53, 54), and mRNA abundance is lower on a per-cell basis for most transcripts in nongrowing bacteria. Nevertheless, the causal relationships between translation, mRNA abundance, RNase expression, and mRNA stability in nongrowing bacteria remain largely untested.

Given the importance of adaptation to energy starvation for mycobacteria, we sought to investigate the mechanisms by which mRNA stability is globally regulated. Here, we show that the global mRNA stabilization response occurs also in Mycobacterium smegmatis—a nonpathogenic model commonly used to study the basic biology of mycobacteria —under hypoxia and carbon starvation. Remarkably, we found that hypoxia-induced mRNA stability is rapidly reversible, with reaeration causing immediate mRNA destabilization even in the absence of protein synthesis. As expected, transcript levels from hypoxic cells were lower on a per-cell basis than those from aerated cultures. However, our data are inconsistent with a model in which mRNA abundance dictates the degradation rate, as has been shown for log-phase E. coli (53) and Lactococcus lactis (54). Instead, our findings support the idea that mRNA stability is rapidly tuned in response to alterations in energy metabolism. This effect does not require the stringent response or changes in abundance of RNA degradation proteins and can be decoupled from growth status.

## RESULTS

### mRNA is stabilized as a response to carbon starvation and hypoxic stress in Mycobacterium smegmatis.

**FIG 1:**
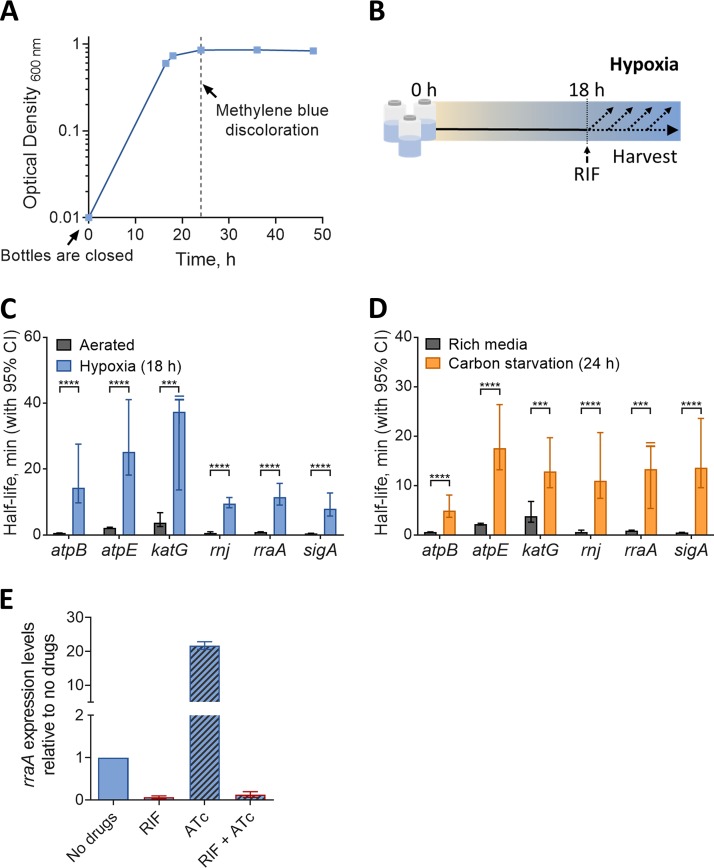
Transcript half-lives are increased in response to hypoxia and carbon starvation stress. (A) Growth kinetics for M. smegmatis under hypoxia using a variation of the Wayne and Hayes model (55), showing OD stabilization at 18 to 24 h. Oxygen depletion was assessed qualitatively by methylene blue discoloration. (B) M. smegmatis was sealed in vials to produce a hypoxic environment, and at 18 h, transcription was inhibited with RIF and samples were collected thereafter. Transcript half-lives for the indicated genes were measured for M. smegmatis mc^2^155 after blocking transcription with 150 μg/ml RIF. (C and D) RNA samples were collected during log-phase normoxia and hypoxia (18 h after closing the bottles) (C) or during log phase in 7H9 supplemented with ADC, glycerol, and Tween 80 (rich medium) or 7H9 with Tyloxapol only (carbon starvation, 24 h) (D). mRNA degradation rates were compared using linear regression (*n = *3), and half-lives were determined by the negative reciprocal of the best-fit slope. Error bars are 95% confidence intervals (CI). ***, *P < *0.001; ****, *P < *0.0001. When a slope of zero was included in the 95% CI (indicating no degradation), the upper limit for half-life was unbounded, indicated by a clipped error bar with a double line. (E) RIF blocks overexpression of an ATc-inducible gene (*rraA*) in hypoxic cultures. Forty hours after the bottles were sealed, cultures were treated with 50 ng/ml ATc and/or 150 μg/ml RIF or the drug vehicle (DMSO) for 1 h. Expression levels (qPCR) are displayed relative to those with no drugs (DMSO treatment). ATc, RIF, and DMSO solutions were degassed prior to addition. Error bars are standard deviations (SD).

The mRNA pools of E. coli and other well-studied bacteria were reported to be globally stabilized during conditions of stress, resulting in increased mRNA half-lives (3–13). Rustad et al. reported a similar phenomenon in M. tuberculosis under hypoxia and cold shock (3). We sought to establish M. smegmatis as a model for study of the mechanistic basis of mRNA stabilization in mycobacteria under stress conditions. We therefore subjected M. smegmatis to hypoxia and carbon starvation and measured mRNA half-lives for a subset of genes by blocking transcription with rifampin (RIF) and measuring mRNA abundance at multiple time points using quantitative PCR (qPCR). We used a variation of the Wayne and Hayes model (55) to produce a gradual transition from aerated growth to hypoxia-induced growth arrest by sealing cultures in vials with defined headspace ratios and allowing them to slowly deplete the available oxygen (Fig. 1A and B). We tested a set of mRNAs that included transcripts with and without leaders, monocistronic and polycistronic transcripts, and transcripts with both relatively short and relatively long half-lives in log phase. We observed that all of the analyzed transcripts had increased half-lives under hypoxia compared to those of log-phase normoxic cultures, and similarly, transcripts were more stable under carbon starvation than in rich media (Fig. 1C and D). Thus, M. smegmatis appears to be a suitable model for investigating the mechanisms of stress-induced mRNA stabilization in mycobacteria. To ensure that the apparent mRNA stabilization was not an artifact of reduced RIF activity in nongrowing cells, we confirmed that RIF indeed blocked transcription in hypoxia-arrested M. smegmatis (Fig. 1E). We noted that transcripts became progressively more stable as oxygen levels dropped and growth ceased; 40 h after sealing the vials, mRNA half-lives were too long to be reliably measured by our methodology. We sought to focus our studies on the mechanisms that underlie the initial mRNA stabilization process during the transition into hypoxia-induced growth arrest. We therefore conducted most of our subsequent experiments 18 to 24 h after sealing the vials, when growth had nearly ceased and transcripts were 9-fold to 25-fold more stable than during log phase. We refer to these conditions as 18-h hypoxia and 24-h hypoxia.

### (p)ppGpp does not contribute to mRNA stabilization under hypoxia or carbon starvation.

**FIG 2:**
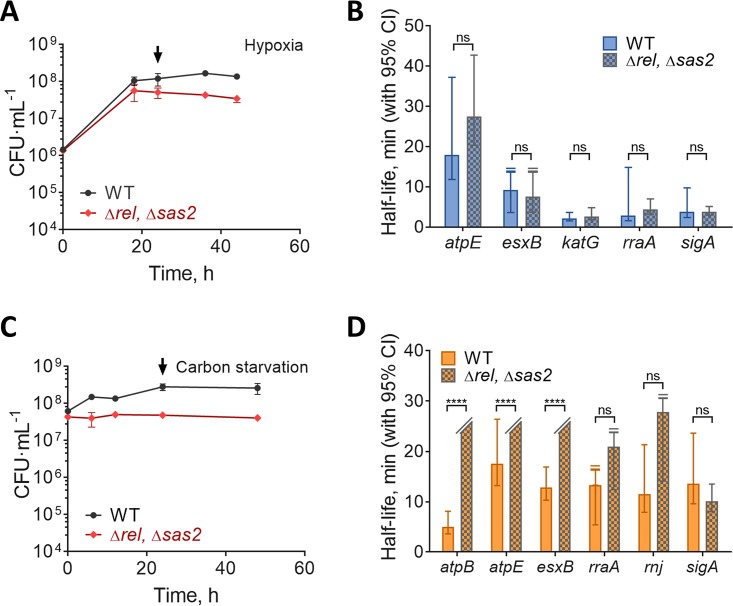
Transcript stabilization in hypoxia and carbon starvation are not dependent on the stringent response. (A) Growth kinetics for M. smegmatis mc^2^155 (wild type [WT]) and Δ*rel* Δ*sas2* strains cultured in 7H9 in flasks sealed at time zero. (B) Transcript half-lives for a set of genes 24 h after sealing of the hypoxia bottles (arrow in panel A). RNA samples were collected after transcription was blocked with 150 μg/ml RIF (degassed). (C) Bacteria were grown to log phase in 7H9 supplemented with ADC, glycerol, and Tween 80 and then transferred to 7H9 supplemented with only Tyloxapol at time zero. (D) Transcript stability for a set of genes 22 h after transfer to carbon starvation medium (arrow in panel C). (A and C) The means and SD of triplicate cultures are shown. (B and D) Half-lives were compared using linear regression analysis (*n *=* *3). Error bars are 95% CI. ****, *P < *0.0001; ns, not significant (*P > *0.05). In cases where no degradation was observed or when the upper 95% CI limit was unbounded, the bar or upper error bar were clipped, respectively.

Given recent reports that (p)ppGpp may directly inhibit the enzymatic activity of the exoribonuclease PNPase (42, 43), we wondered whether mRNA stabilization as observed under carbon starvation and hypoxia is regulated by (p)ppGpp in mycobacteria. We obtained a double mutant strain of M. smegmatis (56) that lacks both genes implicated in the production of (p)ppGpp (*Δrel Δsas2*) and compared the mRNA half-lives of a subset of genes to those of wild-type mc^2^155 under hypoxia, log-phase normoxia, and carbon starvation. The *Δrel Δsas2* strain had a growth defect during adaptation to hypoxia and carbon starvation (Fig. 2A and C), as predicted (57). However, we found no significant decrease in mRNA stabilization in the mutant strain (Fig. 2B and D), indicating that the mRNA stabilization observed under hypoxia and carbon starvation is independent from the stringent response. Interestingly, the mutant strain displayed increased mRNA stabilization for a few transcripts under carbon starvation conditions, which may be an indirect consequence of altered transcription rates (see Discussion).

### Hypoxia-induced mRNA stability is reversible and independent of mRNA abundance.

**FIG 3:**
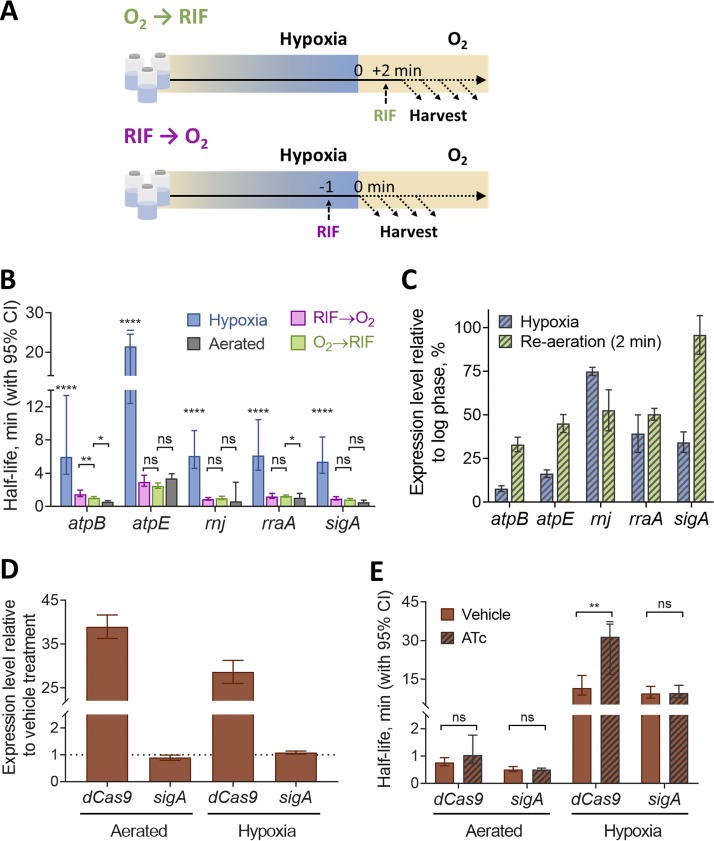
Hypoxia-induced mRNA stability is reversible and independent of mRNA abundance. (A) M. smegmatis was sealed in vials for 18 h to produce a hypoxic environment and then reexposed to oxygen for 2 min before transcription was inhibited with RIF (top) or injected with RIF 1 min prior to opening of the vials and reexposing them to oxygen (bottom). (B) Transcript half-lives for a set of genes are displayed for log-phase normoxia cultures, hypoxia (18 h), and reaeration with RIF added either before or after opening of the vials. Half-lives were compared by linear regression analysis (*n *=* *3). (C) Expression levels of transcripts under hypoxia (18 h) or with a 2-min reaeration relative to the expression levels in log-phase normoxia cultures (percentages). Error bars are SD. (D) Expression levels of transcripts under hypoxia (18 h) or log-phase normoxia after being treated with 200 ng/ml ATc for 1 h or 10 min, respectively, to induce *dCas9* overexpression, relative to the expression levels in cultures treated with an H_2_O vehicle (percentages). Error bars are SD. (E) Transcript half-lives for *dCas9* and *sigA* for log-phase normoxia and hypoxia (18 h) after induction of *dCas9* with ATc or after vehicle treatment as shown in panel D. (B and E) Degradation rates were compared using linear regression (*n = *3), and half-lives were determined by the negative reciprocal of the best-fit slope. Error bars are 95% CI. *, *P < *0.05; **, *P < *0.01; ****, *P < *0.0001; ns, *P > *0.05. RIF added to hypoxic cultures was degassed prior to its addition.

We wondered if the observed stress-induced transcript stabilization could be reversed by restoration of a favorable growth environment. To test this, we prepared 18-h hypoxia cultures, opened the vials, and agitated them for 2 min to reexpose the bacteria to oxygen before blocking transcription with RIF and sampling thereafter (Fig. 3A, top). We found that, for all transcripts tested, half-lives were significantly decreased compared to those observed under hypoxia and similar to those observed in log phase (Fig. 3B). While the mechanisms of stress-induced mRNA stabilization are largely unknown, multiple studies have reported inverse correlations between mRNA abundance and half-life in bacteria (3, 8, 53, 54). mRNA abundance was decreased for most transcripts tested in hypoxia-adapted M. smegmatis. We therefore considered the possibility that the dramatic increase in mRNA degradation upon reexposure to oxygen was triggered by a burst of transcription. Indeed, we found increased expression levels for four of five genes tested after 2 min of reaeration, showing that transcription is rapidly induced upon return to a favorable environment (Fig. 3C). To test the idea that mRNA is destabilized by reaeration as a consequence of a transcriptional burst and/or increased mRNA abundance, we modified our reaeration experiment by blocking transcription with RIF 1 min prior to reaeration (Fig. 3A, bottom). Surprisingly, every transcript tested was destabilized by reaeration despite the absence of new transcription. For most transcripts, the reaeration half-lives were indistinguishable, regardless of whether RIF was added prior to opening the vials or 2 min after (Fig. 3B). Our results therefore do not support the idea that changes in mRNA abundance alone can explain the mRNA stabilization and destabilization observed in response to changes in energy status.

We wanted to further explore whether mRNA abundance alone could influence transcript degradation. We obtained a strain bearing *dCas9* and a nonspecific sgRNA under the control of an anhydrotetracycline (ATc)-inducible promoter (58) and compared the *dCas9* transcript stability under hypoxia and normoxia after ATc induction or at basal levels. We found that despite a 34-fold transcript upregulation following ATc induction, the half-life of *dCas9* mRNA was not significantly different from that of the uninduced control in log phase. Under hypoxia, its 28-fold upregulation was associated with an increase in *dCas9* mRNA half-life compared to in the no-drug control (Fig. 3D and E). Together, our results show that increased mRNA abundance does not necessarily result in a faster decay rate.

### mRNA stability is modulated independently of RNase protein levels.

**FIG 4:**
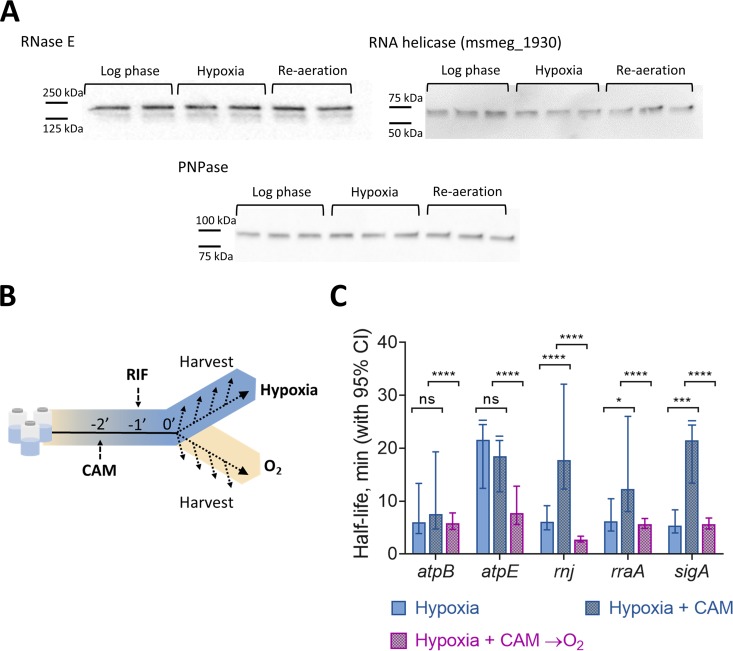
mRNA stability is regulated independently of degradation protein levels. (A) Western blotting for FLAG-tagged RNase E and cMyc-tagged PNPase or RNA helicase (msmeg_1930) in M. smegmatis under log-phase normoxia and hypoxia (18 h) and with a 2-min reaeration. Samples were normalized to the total protein level, and levels were similar on a per-OD basis under all conditions. (B) Translation was inhibited in hypoxic cultures by 150 μg/ml CAM 1 min before the addition of 150 μg/ml RIF. RNA was harvested at time points beginning 2 min after the addition of CAM. (C) Transcript half-lives for samples from hypoxic cultures with the drug vehicle (ethanol), for hypoxic cultures after translation inhibition, and for cultures with 2 min of reaeration after translation inhibition. Degradation rates were compared using linear regression (*n = *3), and half-lives were determined by the negative reciprocal of the best-fit slope. Error bars are 95% CI. ns, *P > *0.05; *, *P < *0.05; ***, *P < *0.001; ****, *P < *0.0001. Drugs and drug vehicles added to the hypoxic cultures were degassed prior to their addition.

Another potential explanation for increased mRNA degradation after reaeration is the upregulation of mRNA degradation proteins, such as RNase E. To assess the role of a sudden burst in protein levels, we used two approaches. First, we constructed strains encoding FLAG-tagged RNase E, cMyc-tagged PNPase, or cMyc-tagged msmeg_1930 (predicted RNA helicase). We determined protein levels by Western blotting in log phase, with 18 h of hypoxia, and after 18 h of hypoxia followed by 2 min of reaeration. Levels of all three of these predicted RNA degradation proteins remained unchanged under the three conditions (Fig. 4A).

Because we do not know all of the proteins that contribute to mRNA degradation in mycobacteria, our second approach was to test the global importance of translation in reaeration-induced mRNA destabilization. We blocked translation with chloramphenicol (CAM) in 18-h hypoxia cultures and then added RIF. Samples were collected for cultures that remained under hypoxia as well as those that were reaerated for 2 min (Fig. 4B). For three of the five genes tested, we found that CAM caused increased mRNA stability under hypoxia. This is consistent with CAM’s mechanism of action and published work (59–61). CAM inhibits elongation by preventing peptidyl transfer (62–64) and causing ribosomal stalling (65). Global stabilization of mRNA pools has been reported when elongation inhibitors, but not initiation inhibitors, are used for example in log-phase cultures of E. coli (65) or in Saccharomyces cerevisiae (66). We hypothesize that stalled ribosomes may increase mRNA stability by masking RNase cleavage sites. However, despite the stabilization caused by CAM itself, we observed mRNA destabilization in response to reaeration (Fig. 4C). These results suggest that reaeration-induced destabilization does not require synthesis of new RNA degradation proteins. Taken together, our data suggest that tuning of protein levels is not the primary explanation for mRNA stabilization during early adaptation to hypoxia.

### mRNA stability is modulated in response to changes in metabolic status.

**FIG 5:**
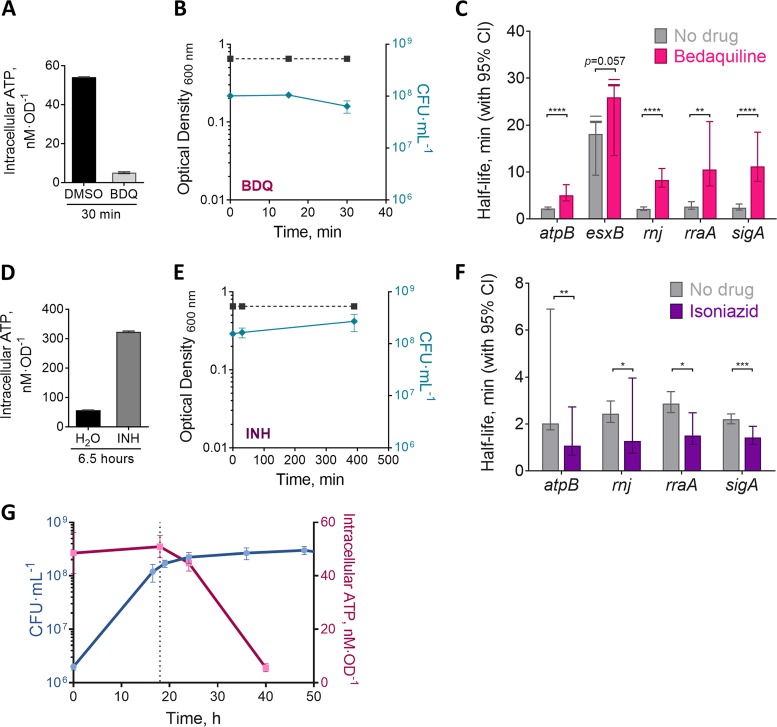
mRNA stability is modulated in response to changes in metabolic status. (A) M. smegmatis was cultured in MMA media for 22 h to OD_600_ 0.8 before being treated with 5 μg/ml BDQ or the vehicle (DMSO) for 30 min. Intracellular ATP was determined using the BacTiter-Glo kit. (B) Growth kinetics for M. smegmatis from panel A in the presence of BDQ. (C) Transcript half-lives for a subset of transcripts collected during intracellular ATP depletion (30 min with BDQ) or at the basal levels (30 min with DMSO). (D) As in panel A, but for M. smegmatis treated with 500 μg/ml INH or the vehicle (H_2_O) for 6.5 h. (E) Growth kinetics for M. smegmatis from panel D in the presence of INH. (F) Transcript half-lives for a subset of transcripts after 6.5 h of INH or vehicle treatment. (G) Growth kinetics for M. smegmatis transitioning into hypoxia, and intracellular ATP levels at different stages. Bottles were sealed at time zero. The dotted line represents the time at which transcript stability analyses were made for the hypoxia (18 h) condition for Fig. 1, 3, and 4. In C and F, half-lives were compared using linear regression analysis (*n *=* *3). Error bars are 95% CI. *, *P < *0.05; **, *P < *0.01; ***, *P < *0.001; ****, *P < *0.0001. ATP was measured in biological triplicate cultures and is representative of at least two independent experiments.

The rapidity of mRNA destabilization following reaeration suggested that mRNA degradation is tightly regulated in response to changes in energy metabolism. We tested this hypothesis by treating log-phase cultures with 5 μg/ml bedaquiline (BDQ), a potent inhibitor of the ATP synthase F_o_F_1_ (67). We used minimal medium that contained acetate as the only carbon source (minimal medium acetate [MMA]) in order to make the respiratory chain the sole source of ATP synthesis. After 30 min of exposure, intracellular ATP levels were reduced by more than 90% compared to levels in cells treated with vehicle (dimethyl sulfoxide [DMSO]), without affecting viability (Fig. 5A and B). We then measured half-lives for a set of transcripts under these conditions. mRNA half-lives were dramatically increased in BDQ-treated cells for most of the genes that we tested (Fig. 5C), indicating that mRNA degradation rates are rapidly altered in response to changes in energy metabolism status.

We then wondered if we could increase mRNA degradation rates by increasing intracellular ATP levels. To test this, we treated M. smegmatis cultures with isoniazid (INH), a prodrug that interferes with the synthesis of mycolic acids and also leads to an accumulation of intracellular ATP due to increased oxidative phosphorylation (68). We exposed M. smegmatis to 500 μg/ml INH for 6.5 h to confirm that we had achieved bacteriostasis (the M. smegmatis doubling time in MMA medium is ∼6 h). As shown in Fig. 5D, INH caused a dramatic increase in intracellular ATP after 6.5 h without affecting cell viability (Fig. 5E). Remarkably, mRNA half-lives were significantly decreased in response to INH (Fig. 5F). To our knowledge, this is the first report of bacterial mRNA being destabilized rather than stabilized in response to a growth-impairing stressor. Our results indicate that mRNA stability is regulated not in response to growth status *per se* but rather to energy metabolism. Although we interpreted ATP levels as a reflection of metabolic status in our INH and BDQ assays, the coupling between mRNA degradation and metabolic status does not appear to be mediated by ATP directly. We measured ATP levels in cultures during the transition to hypoxia-induced growth arrest and found that although ATP levels ultimately decrease under hypoxia as has been reported elsewhere (69, 70), mRNA stabilization precedes the drop in ATP levels (Fig. 5G).

## DISCUSSION

Stressors that cause bacteria to slow or stop growth are typically associated with increased mRNA stability (3–9, 11–13). Many of these same stressors reduce energy availability (69, 70), requiring reductions in energy consumption and optimization of resource allocation. We speculate that the decreased mRNA turnover that accompanies such conditions may be an energy conservation mechanism. For M. tuberculosis, hypoxia can lead to generation of bacterial subpopulations with various degrees of antibiotic tolerance (71–73), facilitating bacterial survival and the acquisition of drug resistance-conferring mutations. Understanding the mechanisms that support the transitions into nongrowing states and subsequent survival in these states is therefore a priority.

The transcriptome of M. tuberculosis has been shown to be stabilized under cold shock and hypoxia (3). Here, we found that M. smegmatis also dramatically stabilized its mRNA in response to carbon starvation and hypoxia. For the first time, to our knowledge, we tested the speed at which this stabilization is reversed in mycobacteria upon restoration of energy availability. Remarkably, mRNAs are rapidly destabilized within minutes of reaeration of hypoxic cultures, suggesting that tuning of mRNA degradation rates is an early step in the response to changing energy availability.

The most straightforward explanation for stress-induced mRNA stabilization seems to be downregulation of the mRNA degradation machinery. Indeed, RNase E is downregulated at the transcript level under hypoxia, and abundance of cleaved RNAs is reduced (74). However, we found that protein levels were unchanged for RNase E and two other proteins predicted to be core components of the mRNA degradation machinery. This is largely consistent with what was reported for M. tuberculosis in a quantitative proteomics study (37), although in that case there was an apparent reduction in levels of an RNA helicase. To address this question in a more agnostic fashion, we tested the importance of translation for transcript destabilization upon reexposure of hypoxic cultures to oxygen. However, reaeration triggered increased transcript degradation even in the absence of new protein synthesis. Regulation of degradation protein levels therefore does not appear to contribute to mRNA stabilization during the initial response to energy stress. However, we found that upon longer periods of hypoxia, transcripts were stabilized to a greater extent than what we observed 18 h after sealing the vials. This suggests that mRNA stabilization progressively increases and may involve multiple mechanisms. As this work focused on the initial transition into hypoxia-induced growth arrest, we cannot discount the possibility that downregulation of the RNA degradation machinery is important for further mRNA stabilization in later hypoxia stages.

Interestingly, we found greater mRNA stabilization in hypoxic cultures treated with CAM. This may result from stalled ribosomes (62, 64) masking RNase cleavage sites. Furthermore, the burst of transcription upon reaeration is blocked by the presence of CAM, causing up to a 4-fold decrease in transcript abundance in the CAM-treated cultures compared to that in the vehicle-treated cultures. This is consistent with the idea that transcription and translation are physically coupled, and blocking translation therefore prevents RNA polymerase from efficiently carrying out transcript elongation, as was reported for E. coli (75–79). The results obtained from the *Δrel Δsas2* strain are also consistent with the idea that the presence of ribosomes affects mRNA stability. Under carbon starvation, this strain had rRNA levels 3-fold higher than those of the WT strain, consistent with the known role of (p)ppGpp in downregulating ribosome biogenesis (80–82). Interestingly, some transcripts were hyperstabilized in the *Δrel Δsas2* strain under carbon starvation, showing virtually no degradation (Fig. 2D). We speculate that the observed mRNA hyperstabilization is caused by increased ribosome abundance, resulting in augmented mRNA-ribosome associations that ultimately protect transcripts from RNases. Alternatively, the increased abundance of rRNA may protect mRNA indirectly by providing alternative targets that compete for interaction with RNases (65).

Transcript abundance has been found to be inversely correlated with mRNA stability in exponentially growing bacteria (3, 8, 53, 54, 83), and experimental manipulation of transcription rates of subsets of genes affected their degradation rates (3, 54). Together, these studies suggest that high rates of transcription inherently increase degradation rates. We report here that during oxygen depletion, transcript levels are reduced in M. smegmatis, which led us to ask whether increased transcript half-lives under stress are a direct result of reduced mRNA levels. However, our data are inconsistent with this idea; mRNA is rapidly destabilized upon reaeration even in the absence of new transcription. We note that one study reported a weak positive correlation between mRNA abundance and stability in log-phase E. coli (12), while another reported mRNA abundance to be positively correlated with stability in carbon-starved Lactococcus lactis (8). Together, these observations and our own suggest that the relationship between mRNA stability and abundance is not yet fully understood and may be fundamentally different in growth-arrested bacteria.

The rapid reversibility of hypoxia-induced mRNA stabilization suggests that mRNA decay and energy metabolic status are closely linked. Consistently with this, we have shown that drug-induced energy stress causes mRNA stabilization but that mRNA decay is increased by a drug that induces a hyperactive metabolic state. To our knowledge, this is the first demonstration that the rate of bacterial mRNA degradation can be decoupled from growth rate and suggests that mRNA decay is controlled by energy status rather than growth rate *per se*. The mechanism by which energy status and mRNA decay are coupled remains elusive; the stringent response is not required, and the stabilization of mRNA during adaptation to hypoxia precedes a decrease in ATP levels. Our data are consistent with two general (nonexclusive) models: mRNA decay may be regulated (i) by protection of transcripts from RNase attack and/or (ii) by direct regulation of the activities of the RNases. Possible explanations that fall within one or both of these frameworks include changes in ribosome occupancy, the presence of other RNA-binding proteins, regulation of the subcellular localization of mRNAs and/or the RNA degradation machinery, and altered degradosome composition. These possibilities should be investigated in future work.

## MATERIALS AND METHODS

### Strains and culture conditions.

Mycobacterium smegmatis strain mc^2^155 or its derivatives (Table 1) were grown in rich medium, Middlebrook 7H9 with albumin dextrose catalase (ADC; final concentrations, 5 g/liter bovine serum albumin fraction V [BSA], 2 g/liter dextrose, 0.85 g/liter NaCl, and 3 mg/liter catalase), 0.2% glycerol, and 0.05% Tween 80, which was shaken at 200 rpm and 37°C to an optical density at 600 nm (OD_600_) of ∼0.8, unless specified otherwise. For hypoxic cultures, we modified the Wayne and Hayes model (55). Bacteria were cultured in 30.5- by 58-mm serum bottles (Wheaton; item 223687, 20 ml) using rich medium and an initial OD_600_ of 0.01. Bottles were sealed with a vial crimper (Wheaton; item W225303) using rubber stoppers (Wheaton; item W224100-181) and aluminum seals (Wheaton; item 224193-01). Oxygen levels were qualitatively monitored using methylene blue.

**TABLE 1:** Strains used and sources

Strain	Characteristics	Reference or source
mc^2^155	M. smegmatis, wild type	85
SS-M_0072	mc^2^155 derivative transformed with plasmid pSS162, containing an ATc-inducible copy of *rraA*	This work
SS-M_0296	mc^2^155 in which the native copy of RNase E (*rne*) is N-terminally tagged with the 6×His-3×FLAG-TEV- 4×Gly linker (CACCACCACCACCACCACGATT ACAAGGATCACGATGGCGATTACAAGGATC ATGACATCGACTATAAGGACGATGACGATA AGGAGAACCTGTACTTCCAGGGCGGCGGCGGC)	This work
SS-M_0412	SS-M_0296 derivative containing a second copy of PNPase (msmeg_2656) with its predicted native promoter and 5′ UTR and N-terminally tagged with the cMyc-4×Gly linker (GAGCAGAAGCTGATC TCGGAAGAGGACCTCGGCGGCGGCGGC) contained within the Giles-integrating plasmid pSS282 (Hyg^r^)	This work
SS-M_0416	SS-M_0296 derivative containing a second copy of the RNA helicase (msmeg_1930) with its predicted native promoter and 5′ UTR and C-terminally tagged with the 4×Gly linker-cMyc (GGCGGCGGCGG CGAGCAGAAGCTGATCTCGGA) contained within a Giles-integrating plasmid pSS285 (Hyg^r^)	This work
Δ*rel*_Msm_ strain	mc^2^155 derivative, *Δrel Δsas2*	56
SS-M_0203	mc^2^155 derivative transformed with plasmid pJR962, containing an ATc regulated *dCas9*	58

For carbon starvation cultures, cells were grown to log phase (OD_600_ = 0.8) in rich medium, pelleted, and rinsed three times with carbon starvation medium (Middlebrook 7H9 with 5 g/liter BSA, 0.85 g/liter NaCl, 3 mg/liter catalase, and 0.05% Tyloxapol) at 4°C and then resuspended in carbon starvation medium to an OD_600_ of 0.8 and incubated at 200 rpm and 37°C.

The RNase E-tagged strain (SS-M_0296) was built using a two-step process. Plasmid pSS250 was derived from pJM1 (84) and contained 1 kb of the sequence upstream and downstream of the *rne* (msmeg_4626) start codon, with the sequence encoding 6×His-3×FLAG-TEV-4×Gly inserted after the start codon. Constructs were built using NEBuilder HiFi (E2621). Integrants were selected with 200 μg/ml hygromycin and confirmed by sequencing. Counterselection with 15% sucrose was followed by PCR screening to identify isolates that underwent second crossovers resulting in loss of the plasmid and retention of tagged *rne*.

The PNPase-tagged strain (SS-M_0412) was built by inserting a second copy of *pnp* (msmeg_2656) with an N-terminal cMyc-4×Gly construct and its predicted native promoter and 5′ untranslated region (UTR) at the Giles phage integration site (plasmid pSS282) into strain SS-M_0296. The RNA helicase-tagged strain (SS-M_0416) was constructed in a similar way but with a C-terminal 4×Gly-cMyc tag on msmeg_1930 (plasmid pSS285).

### RNA extraction and determination of mRNA stability.

Biological triplicate cultures were treated with rifampin (RIF) to a final concentration of 150 μg/ml to halt transcription, and RNA was extracted at various time points thereafter. For exponential and carbon starvation cultures, 7 ml was collected per replicate and time point and snap-frozen in liquid nitrogen (LN2). For hypoxic samples, degassed RIF was injected using a 30-gauge needle, and all samples were sacrificially collected per time point and replicate (7 ml) and snap-frozen in LN2 within 6 s of unsealing.

Samples were stored at −80°C and thawed on ice immediately before RNA extraction. Cells were pelleted at 4°C, resuspended in 1 ml TRIzol (Invitrogen), transferred to 2-ml disruption tubes (OPS Diagnostics; 100-μm zirconium lysing matrix, molecular grade), and lysed using a FastPrep-24 5G instrument (MP Biomedical) (3 cycles of 7 m/s for 30 s, with 2 min on ice between cycles). Chloroform (300 μl) was added, samples were centrifuged for 15 min at 21,130 × *g* and 4°C, and RNA was recovered from the aqueous layer and purified with a Direct-zol RNA miniprep kit according to the manufacturer’s instructions with an in-column DNase treatment. Agarose gels were used to verify RNA integrity.

For cDNA synthesis, 600 ng of total RNA was mixed with 0.83 μl 100 mM Tris, pH 7.5, and 0.17 μl 3-mg/ml random primers (NEB) in 5.25 μl, denatured at 70°C for 10 min, and snap-cooled. Reverse transcription was performed for 5 h at 42°C using 100 U ProtoScript II reverse transcriptase (NEB), 10 U RNase inhibitor (murine; NEB), a mix containing 0.5 mM each deoxynucleoside triphosphate (dNTP), and 5 mM dithiothreitol (DTT) in a final volume of 10 μl. RNA was degraded with 5 μl each 0.5 mM EDTA and 1 N NaOH at 65°C for 15 min, followed by 12.5 μl of 1 M Tris-HCl, pH 7.5. cDNA was purified using the MinElute PCR purification kit (Qiagen) according to the manufacturer’s instructions. mRNA abundance (*A*) over time (*t*) was determined for different genes (primers in Table 2) by quantitative PCR (qPCR) using iTaq SYBR green (Bio-Rad) with 400 pg of cDNA and 0.25 μM each primer in 10-μl reaction mixtures, with 40 cycles of 15 s at 95°C and 1 min at 61°C (Applied Biosystems 7500). Abundance was expressed as the negative threshold cycle (–*C_T_*) [reflecting the log_2_*A*(*t*)]. Linear regression was performed on *–C_T_* values versus time where the negative reciprocal of the best-fit slope estimates mRNA half-life (see Text S1 and Fig. S1 in the supplemental material). In many cases, the decay curves were biphasic, with a rapid period of decay followed by a period of slow or undetectable decay. In these cases, only the initial, steeper slope was used for calculation of half-lives.

**TABLE 2:** Primers for qPCR

Primer name (reference)	Gene (locus tag)	Directionality	Sequence (5′→3′)
SSS903	*atpB* (msmeg_4942)	Forward	TGTTCGTGTTCGTCTGCTAC
SSS904	*atpB* (msmeg_4942)	Reverse	CGGCTTGGCGAGTTCTT
SSS909	*atpE* (msmeg_4941)	Forward	GGGTAACGCGCTGATCTC
SSS910	*atpE* (msmeg_4941)	Reverse	GAAGGCCAGGTTGATGAAGTA
SSS1241	*dCas9*	Forward	GACAAGTCGAAGTTCCTGATGTA
SSS1242	*dCas9*	Reverse	GATCTGCTTGTTCGGGTAGTT
SSS537	*esxB* (msmeg_0065)	Forward	GGTGAGGACACAGGGAAATAAG
SSS538	*esxB* (msmeg_0065)	Reverse	CGGAGATGCGCTCGAAAT
SSS856	*katG* (msmeg_6384)	Forward	GGCCCAATCAGCTCAATCT
SSS857	*katG* (msmeg_6384)	Reverse	CGGACCGGTAGTCGAAATC
SSS706	*rnj* (msmeg_2685)	Forward	TCATCCTCTCATCGGGTTTC
SSS707	*rnj* (msmeg_2685)	Reverse	TTCGCGCTCAACCTTCT
SSS697	*rraA* (msmeg_6439)	Forward	AACTACGGCGGCAAGAT
SSS698	*rraA* (msmeg_6439)	Reverse	GTCGAGAGGATCGACTTCAG
JR273 (58)	*sigA* (msmeg_2758)	Forward	GACTACACCAAGGGCTACAAG
JR274 (58)	*sigA* (msmeg_2758)	Reverse	TTGATCACCTCGACCATGTG

10.1128/mBio.00957-19.1 TEXT S1 Additional details about the methodology used to measure mRNA half-lives are provided. Download Text S1, DOCX file, 0.02 MB. Copyright © 2019 Vargas-Blanco et al. 2019 Vargas-Blanco et al. This content is distributed under the terms of the Creative Commons Attribution 4.0 International license.

10.1128/mBio.00957-19.1 FIG S1 mRNA decay curves for a sample gene, *rraA*. The *x* axis denotes the time after transcription was blocked by addition of RIF. (A) –*C_T_* versus time data for *rraA*, giving a half-life estimate of 0.935 min. (B) Estimated mRNA abundance for *rraA* relative to the time of RIF addition, giving a half-life estimate of 0.935 min. Download FIG S1, TIF file, 2.2 MB. Copyright © 2019 Vargas-Blanco et al. 2019 Vargas-Blanco et al. This content is distributed under the terms of the Creative Commons Attribution 4.0 International license.

### mRNA stability during reaeration and translational inhibition.

Translation was halted by 150 μg/ml chloramphenicol, rifampin was added 1 min later, and samples were collected starting 1 min after that. For reaeration experiments, 18-h hypoxia cultures were opened and the contents transferred to 50-ml conical tubes, and triplicate samples were taken 2, 7, 12, 17, and 32 min after we opened the bottles and snap-frozen in LN2. Rifampin was added either 1 min before (transcription inhibition during hypoxia) or 2 min after (transcription inhibition after reaeration) we opened the bottles.

### BDQ and INH treatments.

Cultures were grown to an OD_600_ of ∼1.0 in rich medium, rinsed twice in minimal medium acetate (MMA) wash (final concentrations, 0.5 g/liter l-asparagine, 1 g/liter KH_2_PO_4_, 2.5 g/liter Na_2_HPO_4_, 0.5 g/liter MgSO_4_·7H_2_O, 0.5 mg/ml CaCl_2_, 0.1 mg/ml ZnSO_4_, 0.1% CH_3_COONa, 0.05% tyloxapol, pH 7.5) at 4°C, resuspended in MMA (MMA wash plus 50 mg/liter ferric ammonium citrate) to an OD_600_ of 0.07, and grown for 24 h to an OD_600_ of ∼0.8. To remove the extracellular ATP, 30 min before drug treatment, cells were pelleted and rinsed in prewarmed MMA wash, resuspended in prewarmed MMA, and returned to the incubator. Bedaquiline (BDQ), isoniazid (INH), or their vehicles were added to final concentrations of 5 μg/ml (BDQ) or 500 μg/ml (INH). Samples were taken 30 min after the addition of BDQ or 6.5 h after the addition of INH for half-life and ATP determinations.

For half-life measurements, BDQ cultures were sampled 0, 3, 6, 9, 12, 15, and 21 min after addition of RIF, and INH cultures were sampled 0, 4, 8, and 12 min after addition of RIF. RNA extractions were performed as described above, with the following modifications: cell disruption was performed using 2-ml tubes prefilled with lysing matrix B (MP Biomedical) and 3 cycles of 10 m/s for 40 s, RNA was recovered from the aqueous layer by isopropanol precipitation and resuspension in H_2_O, and samples were treated with 5 U of Turbo DNase (Ambion) in the presence of 80 U of RNase inhibitor, murine (NEB) for 1 h at 37°C with agitation. RNA was purified with an RNeasy minikit (Qiagen) according to the manufacturer’s specifications.

### Intracellular ATP estimation.

ATP was estimated by BacTiter-Glo (Promega). For BDQ or INH treatments, 1 ml of culture was pelleted at ∼21°C for 1 min at 21,130 × *g*, the supernatant removed, and cells resuspended in 1 ml of prewarmed MMA containing BDQ, INH, or vehicle to match the prior treatment conditions. Immediately after, 20-μl samples were transferred to a white 384-well plate (Greiner bio-one) containing 80 μl of BacTiter-Glo reagent and mixed for 5 min at room temperature. Luminescence was measured in a Victor^3^ plate reader (PerkinElmer) (intracellular ATP). We included controls for the supernatant collected (extracellular ATP), media plus drug/vehicle (background), and ATP standards for constructing standard curves.

To estimate intracellular ATP in normoxia and hypoxia cultures, 20-μl samples were collected at 37°C and immediately combined with the reagent to measure total ATP (intracellular plus extracellular). From the same cultures, 1-ml samples were syringe filtered (PES; 0.2 μm) and the filtrate was combined with the reagent to measure extracellular ATP. Luminescence was measured as described above. Intracellular ATP was calculated by subtracting the extracellular ATP values from the total ATP values. Hypoxia samples were sacrificially harvested per time point/replicate and combined with the reagent in <6 s.
