# Supplementary material for: mRNA Degradation Rates Are Coupled to Metabolic Status in Mycobacterium smegmatis
Source: mBio. 2019 Jul 2;10(4):e00957-19. doi: 10.1128/mBio.00957-19 (PMC6606801; doi:10.1128/mBio.00957-19)
Supplement: TEXT S1 [file mBio.00957-19-s0001.docx]

**Supplemental Methods:**

mRNA half-life experiments were conducted using purified RNA that was reverse-transcribed into cDNA. cDNA samples were purified as described in Methods, which allowed us to quantify cDNA concentrations and use the same amount of cDNA in each qPCR reaction. Because total RNA is composed mostly of rRNA, our cDNA was also primarily composed of rRNA sequences. rRNA is much more stable than mRNA and does not appreciably decay over the RIF treatment periods that we used for measuring mRNA decay rates, as evidenced by our observations that total RNA per OD unit or per CFU was unaltered during these treatment periods. Hence, we assumed that as mRNA decays, its abundance will change relative to total RNA abundance. When performing qPCR for a gene of interest, we therefore interpreted the cycle threshold (C_T_) as a reflection of the log_2_ abundance of that transcript relative to total RNA levels. By performing qPCR on a defined amount of carefully quantified cDNA, we were able to measure abundance of specific mRNAs relative to total RNA content which consists mostly of stable rRNA.

Half-lives were estimated as the negative reciprocal of the slope of the regression line for log_2_ mRNA abundance versus time. If we assume that the overall abundance of mRNA at time *t* (min) after transcription is arrested, *A*(*t*), follows an exponential decay curve, then

$A(t)=A(0){\cdot2}^{-t/\tau}$,

where *A*(0) is the initial abundance of mRNA and *τ* is the half-life (min). Taking the logarithm (base 2) of both sides gives

$$\log_{2} A(t)=\log_{2} A(0)-\frac{t}{\tau} .$$

To measure *A*(*t*), we freeze samples after *t* minutes and determine the cycle threshold, *C­_T_* (*t*), which is the number of duplications required to reach a fixed threshold (*A**) when starting with *A*(*t*). Thus,

$$A^{*}=A\left( t \right)\cdot2^{C_{T}\left( t \right)}$$

$$A(t)=A^{*}\cdot2^{-C_{T}\left( t \right)}$$

Substituting into both sides of the equation for log_2_ abundance over time,

$$\log_{2} \left( A^{*}\cdot2^{-C_{T}\left( t \right)} \right)=\log_{2} \left( A^{*}\cdot2^{-C_{T}\left( 0 \right)} \right)-\frac{t}{\tau}$$

$$\log_{2} A^{*}-C_{T}(t) =\log_{2} A^{*}-C_{T}(0)-\frac{t}{\tau}$$

$$-C_{T}(t)={-C}_{T}(0)-\frac{t}{\tau} .$$

Thus, when –*C_T_* (which represents abundance on a log_2_ scale) is plotted as a function of time, the half-life *τ* = -1/slope. An example is shown in Fig. S1 in the supplemental material.
