# Supplementary figures and images for: mRNA Degradation Rates Are Coupled to Metabolic Status in Mycobacterium smegmatis
Source: mBio. 2019 Jul 2;10(4):e00957-19. doi: 10.1128/mBio.00957-19 (PMC6606801; doi:10.1128/mBio.00957-19)

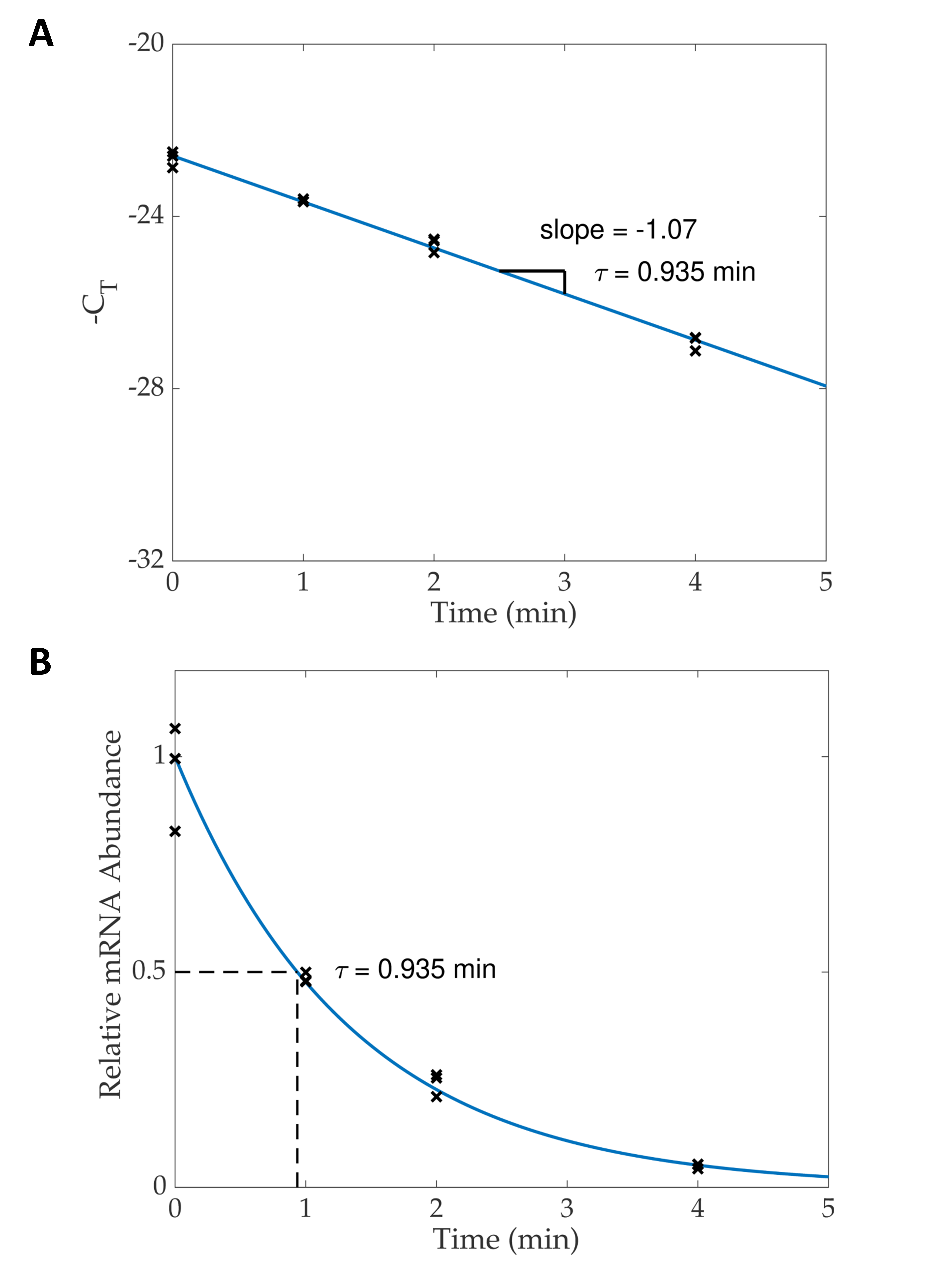

Supplement: FIG S1 [file mBio.00957-19-sf001.tif]
